# FROM LEFT TO RIGHT. PARA-AORTIC LYMPH NODES SAMPLING DURING PANCREATODUODENECTOMY FOR PANCREATIC CANCER

**DOI:** 10.1590/0102-672020230054e1772

**Published:** 2023-11-13

**Authors:** Gabrielle Stevenin, Clémence Guyard, Renato Micelli Lupinacci

**Affiliations:** 1 Ambroise Paré Hospital, Department of Digestive and Oncologic Surgery – Boulogne-Billancourt, France; 2 Paris Saclay University, Versailles St-Quentin-en-Yvelines – Montigny-le-Bretonneux, France.

**Keywords:** Pancreatic Neoplasms, Neoplasm Staging, Lymph Node Excision, Pancreaticoduodenectomy, Neoplasias Pancreáticas, Estadiamento de Neoplasias, Excisão de Linfonodo, Pancreaticoduodenectomia

## Abstract

**BACKGROUND:**

Para-aortic lymph nodes involvement in pancreatic head cancer has been described as an independent adverse prognostic factor. To avoid futile pancreatic resection, we systematically perform para-aortic lymphadenectomy as a first step.

**AIMS:**

To describe our technique for para-aortic lymphadenectomy.

**METHODS:**

A 77-year-old female patient, with jaundice and resectable pancreatic head adenocarcinoma, underwent pancreaticoduodenectomy associated with infracolic lymphadenectomy.

**RESULTS:**

The infracolic anterior technique has two main advantages. It is faster and prevents the formation of postoperative adhesions, which can make subsequent surgical interventions more difficult.

**CONCLUSIONS:**

We recommend systematic para-aortic lymphadenectomy as the first step of pancreaticoduodenectomy for pancreatic head adenocarcinoma by this approach.

## RESUMO

**RACIONAL:**

O envolvimento dos gânglios linfáticos para-aórticos no câncer da cabeça do pâncreas tem sido descrito como um fator prognóstico adverso independente. Para evitar a ressecção pancreática inútil, realizamos sistematicamente linfadenectomia para-aórtica.

**OBJETIVOS:**

Descrever a técnica de linfadenectomia para-aórtica.

**MÉTODOS:**

Paciente do sexo feminino, 77 anos, com quadro de icterícia e adenocarcinoma da cabeça do pâncreas ressecável, submetida à duodenopancreatectomia associada à linfadenectomia infracólica.

**RESULTADOS:**

Esta técnica anterior infracólica tem duas vantagens principais: é mais rápida e evita a formação de aderências pós-operatórias, o que pode dificultar as intervenções cirúrgicas subsequentes.

**CONCLUSÕES:**

Recomendamos a linfadenectomia para-aórtica sistemática como o primeiro passo da duodenopancreatectomia para o adenocarcinoma da cabeça do pâncreas por esta abordagem.

## INTRODUCTION

Pancreatic cancer remains a major therapeutic challenge despite medical advances with a 12% 5-year survival rate^9^. As opposed to most other cancer types, incidence and death rates for pancreatic cancer continue to rise and estimates put pancreatic cancer as the second leading cause of cancer deaths in Western countries before 2030^9^. The biological heterogeneity and the complexity of patients with pancreatic cancer require a personalized and multidisciplinary approach to choosing the most efficient treatment for each clinical situation^2^.

Metastasis to para-aortic lymph nodes (PALN) in pancreatic head cancer is common, with the incidence ranging from 12 to 28%^3,10,11,13-15^. PALN has been found to be an independent adverse prognostic factor^5,11,12^. In this context, considering metastatic PALN as a contraindication to pancreaticoduodenectomy (PDD) in up-front resectable pancreatic cancer is a source of fiery debate^9-11^. Some studies have shown that patients with metastatic PALN could still benefit from radical PDD compared to the double bypass procedure^7^ and benefit from modern chemotherapy regimens^3,6^.

The aim of this article is to describe our technique for PALN sampling. We perform it systematically from the “left” as the first step of our PDD for pancreatic head cancer. We strongly prefer this approach over the “classical approach” which demands a complete Kocher maneuver.

## METHODS

To illustrate our technique, we describe the first step of a PDD for pancreatic head adenocarcinoma in a 77-year-old female patient who presented with jaundice. Computed tomography (CT) scan and magnetic resonance imaging (MRI) showed the presence of a well-defined tumor arising from the pancreatic head with upstream dilatation of the common biliary duct and the main pancreatic duct. After a multidisciplinary discussion, the patient was recommended for upfront surgery. Postoperative course was uneventful. The length of hospital stay was eight days. Final pathology demonstrated a 1,5 cm pancreatic ductal adenocarcinoma pT1N2 (5/33) M0 R0, with negative surgical margins.

### Surgical technique

The operation begins with the patient in supine reverse Trendelenburg position (30°). The surgeon stands to the right of the operating table. We usually perform a midline laparotomy but a transverse incision is sometimes dictated by the patient's anatomy and the surgeon's discretion^8^. We reach the aortocaval space through an infracolic anterior approach lateral to the mesenteric vessels.

**Figure 1:**
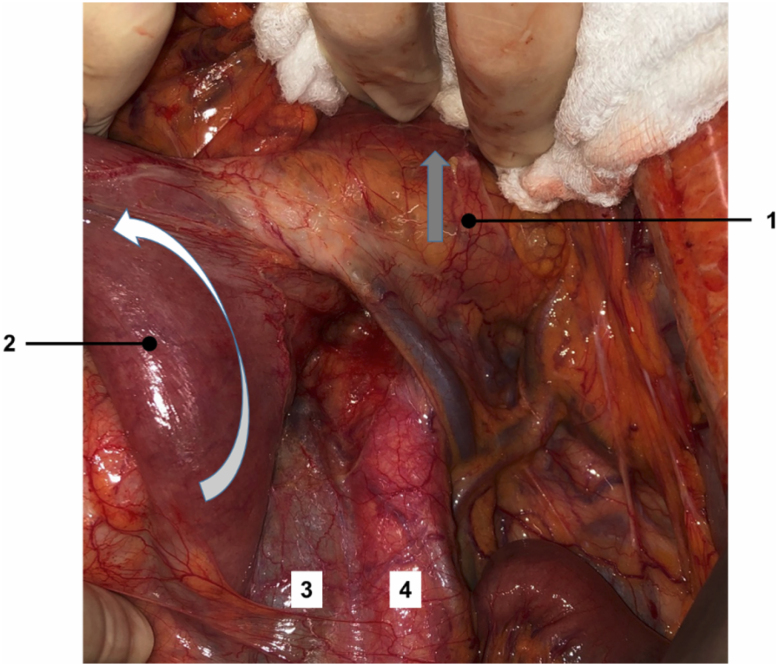
Gray arrow indicates the direction of traction of the transverse mesocolon (1). White arrow indicates the traction of the first jejunal loop (2) to achieve correct exposure of the inferior vena cava (3) and the aorta (4).

**Figure 2:**
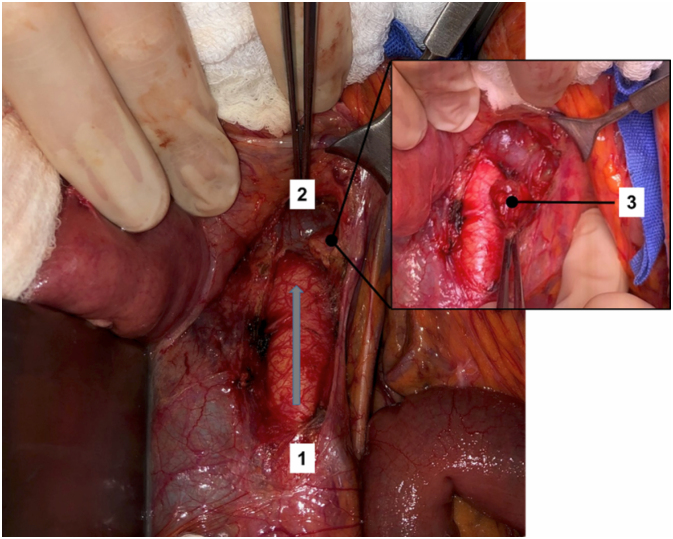
Gray arrow indicates the incision of the retroperitoneal anterior to the aorta. It begins usually at the level of the inferior mesenteric artery (1) and extends cranially until the left renal vein (2) is identified and fully exposed. A latero-aortic infrarenal lymph node is harvested (3).

**Figure 3:**
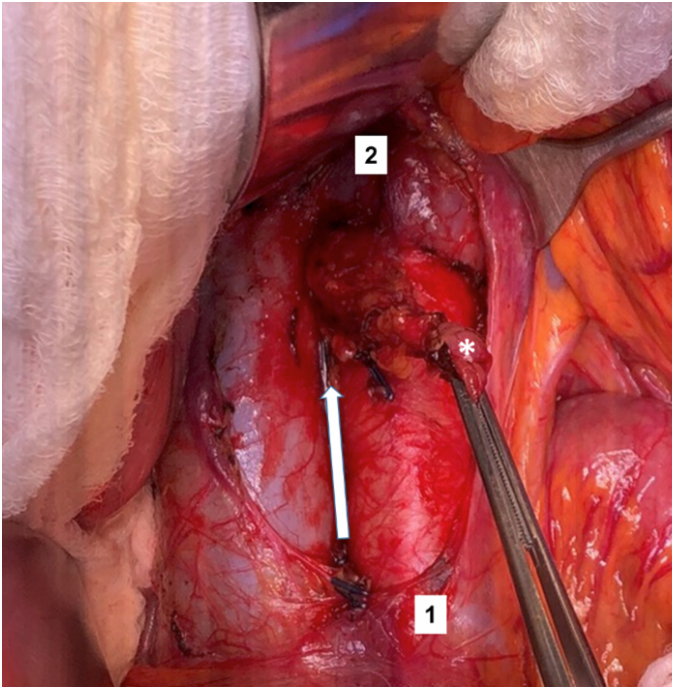
Incision of the retroperitoneal anterior to the inferior vena cava. Dissection of the interaortocaval nodes (*) begins (white arrow) at the level of the inferior mesenteric artery (1) and extends cranially to the inferior border of the left renal vein (2).

**Figure 4:**
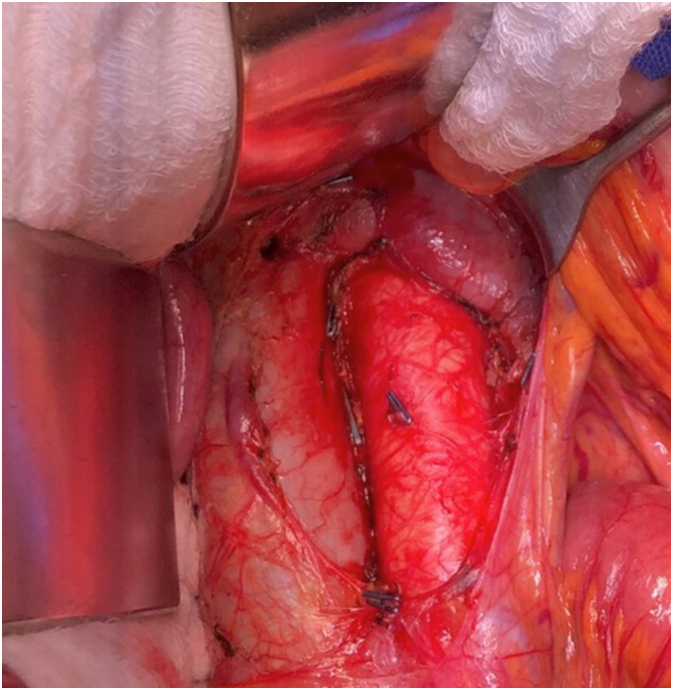
Final aspect of the surgical field after para-aortic lymph nodes sampling.

- Step 1: The transverse colon is retracted upward and the intestinal bundle is retracted laterally to the right, allowing for identification of the Treitz ligament. Dissection begins with the release of the duodenojejunal angle, which allows the mobilization of the 4^th^ portion of the duodenum to the right and exposure of the anterior wall of the inferior vena cava and the infrarenal aorta (Figure 1).
- Step 2: The incision over the retroperitoneum anterior to the aorta provides exposure of the anterior aortic wall. The extension of the retroperitoneal incision cranially allows the left renal vein to be identified and fully exposed, which marks the upper limit for the aortocaval space dissection (Figure 2).
- Step 3: An incision of the retroperitoneal anterior to the inferior vena cava is performed. The dissection of the aortocaval lymph nodes begins at the level of the inferior mesenteric artery and extends cranially to the inferior border of the left renal vein. We use surgical clips and LigaSure™ to obtain hemostasis and lymphostasis (Figures 3 and 4).

## RESULTS

During a first step of PDD for pancreatic adenocarcinoma, between January 2021 and December 2022, 48 patients had PALN. The median operative time (for PALN sampling) was 17 (range 12–31) minutes. The median number of PALN analyzed per patient was 4 (range 2–13). Seven (14,5%) patients had positive PALN in frozen section analysis. One patient had grade A chyle leak and two (4,2%) had grade B chyle leak^1^ successfully treated with dietary restriction, total parenteral nutrition, octreotide, and maintenance of surgical drains as previously published^4^.

## DISCUSSION

Metastasis to PALN is considered an independent adverse prognostic factor^5,11^. However, there is no consensus on the dissection of a 16b1 group^16^. Recent studies showed that PALN involvement might not affect survival in patients who complete the full curative strategy (adequate systemic treatment and radical surgery)^6,8,15^. Thus, we systematically perform PALN dissection with frozen-section analysis in order to guide a personalized surgical approach, as follows:

1. In low-risk patients with resectable tumors, we keep on with the pancreas resection, regardless of the 16b1 status;
2. In patients with borderline/locally advanced tumors, intervention is stopped and systemic chemotherapy is rapidly resumed;
3. In high-risk patients with resectable tumors who underwent upfront surgery, the intervention is stopped and we start systemic chemotherapy with FOLFIRINOX as soon as possible. After eight cycles, we evaluate tumor response by CT scan, MRI, and the cancer antigen (CA)19–9. In case of good tumor response, we propose the return to the operating room for pancreatic resection after multidisciplinary discussion.

## CONCLUSIONS

We strongly suggest pancreatic surgeons approach the aortocaval regions with this infracolic anterior technique, which, in our experience, has two main advantages. First, it is faster than and at least as secure as the “classical approach.” Second, potential postoperative adhesions do not make any further intervention more laborious.
